# The Role of Dominant Lesion Site and SUVmax in the Depth of PSA Response in Metastatic Hormone-Sensitive Prostate Cancer

**DOI:** 10.3390/biomedicines14102160

**Published:** 2026-09-24

**Authors:** Güner Akgüner, Ayşegül İlhan Güleşen, Özgen Ahmet Yıldırım

**Affiliations:** Department of Medical Oncology, Ankara Etlik City Hospital, Ankara 06170, Türkiyeozgenayildirim@gmail.com (Ö.A.Y.)

**Keywords:** prostate cancer, hormone-sensitive, PSMA PET/CT, SUVmax, bone metastasis, PSA nadir, androgen receptor pathway inhibitor

## Abstract

**Background/Objectives**: Depth of prostate-specific antigen (PSA) response is prognostic in metastatic hormone-sensitive prostate cancer (mHSPC), and the SUVmax of the most avid lesion is a readily available parameter on staging PSMA PET/CT. Uptake intensity and the anatomical site of that lesion are, however, distinct features. This study evaluated their associations with the depth of PSA response. **Methods**: In this retrospective single-centre study of men receiving androgen deprivation therapy plus an androgen receptor pathway inhibitor (ARPI), the SUVmax and anatomical site of the dominant lesion were derived from baseline ^68^Ga-PSMA-11 PET/CT. The primary outcome was a PSA nadir < 0.2 ng/mL, analysed using logistic regression with sequential adjustment and within strata defined by disease burden. Progression-free survival (PFS) was a secondary outcome. **Results**: Among 100 men with evaluable baseline PET/CT, SUVmax was associated with neither PSA response depth nor PFS in any parameterisation examined. A deep PSA response was achieved by 72%, 52% and 29% of patients with prostate-, node- and bone-dominant disease (trend *p* < 0.001), with median nadirs of 0.08, 0.19 and 0.50 ng/mL. Among patients with bone metastases, a deep response occurred in 29% with bone-dominant disease and 57% with a dominant lesion elsewhere (*p* = 0.016). The association persisted across sequentially adjusted models (OR range 0.22–0.33) and within high-volume disease (*p* = 0.008). Under a six-month landmark, deep PSA response was associated with longer PFS (HR 0.10, 95% CI 0.01–0.42), whereas bone dominance was not. **Conclusions**: The site of the dominant PSMA PET/CT lesion, but not its SUVmax, was associated with the depth of PSA response in ARPI-treated mHSPC. These findings require prospective validation.

## 1. Introduction

Prostate cancer is the malignancy most often diagnosed in men and one of the leading causes of male cancer death [1]. Since the demonstration that prostate cancer is androgen-dependent, suppression of testosterone to castrate levels has anchored treatment of metastatic disease [2], although resistance eventually emerges through restored androgen receptor signalling and related mechanisms [3].

The therapeutic landscape has been transformed over the past decade. Docetaxel added to androgen deprivation therapy (ADT) was the first strategy shown to extend survival in the hormone-sensitive setting [4,5], and the androgen receptor pathway inhibitors (ARPIs) abiraterone, enzalutamide and apalutamide each did the same when combined with ADT [6,7,8]. Triplet regimens combining ADT, docetaxel and an ARPI have since improved outcomes further [9,10].

This increasing therapeutic efficacy has also shifted the focus of response assessment. A decline of at least 50% in prostate-specific antigen (PSA) remains incorporated into standard response criteria [11]; however, such responses are increasingly common with contemporary combination therapies, potentially limiting the discriminatory value of this conventional threshold. Accordingly, greater attention has been directed toward the depth of PSA response. A PSA nadir below 0.2 ng/mL has been associated with improved survival in long-term analyses of CHAARTED [12] and post hoc analyses of LATITUDE and TITAN [13,14].

Prostate-specific membrane antigen (PSMA)-targeted PET has emerged as a highly sensitive imaging modality for staging prostate cancer, frequently identifying metastatic disease not detected by conventional imaging [15]. Among the quantitative parameters derived from PSMA PET, the SUVmax of the most avid lesion is readily available and commonly reported in routine clinical practice. Volumetric measures such as PSMA-derived total tumour volume carry prognostic information [16] but require dedicated post-processing that is less frequently performed outside research settings. PSMA-PET-CT-derived parameters have shown prognostic associations in patients with castration-resistant prostate cancer undergoing PSMA-targeted radioligand therapy [17]; however, their prognostic relevance in the hormone-sensitive setting remains less well defined.

PSMA PET-CT provides two distinct dimensions of information: the intensity of tracer uptake, quantified by SUVmax, and the anatomical location of the most avid lesion, which is not captured by that numerical value. Standardised reporting frameworks document lesion location systematically [18], yet this information is seldom treated as a prognostic variable in its own right. Metastatic site has been associated with overall survival in castration-resistant disease [19], and this may be particularly relevant for bone metastases, as the bone microenvironment is not merely a passive site of tumour dissemination but provides a biologically active niche capable of supporting prostate cancer cell survival [20,21].

The site of the dominant lesion should also be distinguished from the mere presence of metastases at a given anatomical site. In particular, the presence of bone metastases and the identification of bone as the site of the SUVmax-dominant lesion represent conceptually different features: the former describes metastatic distribution, whereas the latter identifies the anatomical location of the most PSMA-avid disease. Whether this distinction provides additional information regarding treatment response remains uncertain.

Accordingly, this study evaluated the SUVmax and anatomical site of the dominant lesion as separate imaging features and examined their associations with the depth of PSA response in patients with mHSPC treated with ARPI-based therapy. We further explored the relationship between PSA response depth and subsequent disease progression. Given the retrospective and hypothesis-generating nature of the study, the analyses were interpreted with appropriate adjustment for multiple testing.

## 2. Materials and Methods

### 2.1. Study Design and Setting

This was a retrospective, single-centre cohort study of men with metastatic hormone-sensitive prostate cancer (mHSPC) treated with an ARPI in combination with ADT. The study was conducted in accordance with the principles of the Declaration of Helsinki. The study protocol was approved by the Ankara Etlik City Hospital Scientific Research Evaluation and Ethics Committee (approval number AEŞH-BADEK-2025-0005, dated 8 January 2025), which waived the requirement for individual informed consent due to the retrospective design and use of de-identified data.

### 2.2. Eligibility Criteria

Consecutive men treated at the Department of Medical Oncology, Ankara Etlik City Hospital, between November 2022 and November 2024 were screened for eligibility. Inclusion criteria were a diagnosis of metastatic prostate cancer that was hormone-sensitive at the initiation of systemic therapy; treatment with ADT in combination with an ARPI (abiraterone, enzalutamide, or apalutamide), either as doublet therapy or as part of a triplet regimen including docetaxel; a documented baseline PSA measurement; and at least one on-treatment PSA measurement within the first six months of therapy.

Patients were excluded if the available clinical records were insufficient to assess the depth of PSA response, if they were lost to institutional follow-up, or if they had a concurrent second primary malignancy. Of 158 patients screened, 53 were excluded: 32 because of insufficient clinical data, 15 because of loss to follow-up, and 6 because of a concurrent second primary malignancy. The final study cohort therefore comprised 105 patients (Figure 1). Of these, 100 had an evaluable baseline ^68^Ga-PSMA-11 PET/CT and constituted the population for the imaging-based analyses.

### 2.3. Variable Definitions

Data were abstracted from the institutional electronic medical records. Metastatic burden was classified according to the CHAARTED high- versus low-volume definition [4] and the LATITUDE high- versus low-risk definition [6], and tumour grade according to the ISUP grade group. Bone, nodal, and visceral metastatic involvement was recorded independently, together with de novo versus metachronous presentation and prior local therapy. Baseline haemoglobin, albumin, lactate dehydrogenase (LDH), and complete blood count parameters were recorded at or immediately before the initiation of systemic therapy. For survival analyses, continuous laboratory variables were dichotomised at the cohort median.

PSA levels were obtained from measurements performed during routine clinical follow-up. Baseline PSA was defined as the value recorded at or immediately before the initiation of systemic therapy, and the six-month PSA nadir was defined as the lowest PSA value recorded within the first six months after treatment initiation. A deep PSA response was defined as a six-month PSA nadir < 0.2 ng/mL. Conventional PSA50 and PSA90 response categories were also calculated for descriptive purposes. On-treatment PSA was measured at approximately one, three and six months in accordance with departmental practice, and the six-month nadir was defined as the lowest of these values. The exact interval between measurements was not recorded.

### 2.4. Imaging

Baseline ^68^Ga-PSMA-11 PET/CT was performed as part of routine clinical staging within one month before the initiation of systemic therapy, and in all cases before the first dose of ADT, so that no scan was obtained under the influence of androgen suppression. Patients received a weight-adjusted activity of 1.8–2.2 MBq/kg and were imaged 60 min after injection on a Discovery IQ PET/CT scanner (GE HealthCare, Milwaukee, WI, USA). Images were acquired in three-dimensional mode from the vertex to the proximal thigh at three minutes per bed position. Low-dose CT (120–140 kVp, 50 mAs) was acquired for attenuation correction, and PET images were reconstructed using the VUE Point HD algorithm with four iterations and twelve subsets. Acquisition and reconstruction followed standard departmental clinical protocols rather than a research protocol; parameters were not standardised across the study period and SUV values were not cross-calibrated.

The dominant lesion was defined as the lesion with the highest SUVmax, and its anatomical site was classified as prostate, lymph node, bone, or visceral/other according to the clinical imaging report. Because visceral and other dominant sites were uncommon, site-based analyses focused primarily on prostate-, node-, and bone-dominant disease, with an additional comparison between bone-dominant and non-bone-dominant disease.

Follow-up PSMA PET/CT was available only in a subset of patients, and quantitative SUVmax measurements were not consistently available; therefore, longitudinal metabolic response was not evaluated.

To assess the reproducibility of the two imaging variables, all baseline scans were independently re-read by a second nuclear medicine physician who was blinded to the original report, to the original SUVmax and dominant lesion site, and to all clinical and outcome data. Both readers were nuclear medicine physicians with more than five years of experience in PSMA PET/CT reporting. The second reader evaluated the original images rather than the clinical reports and recorded, for each scan, the SUVmax of the single most avid lesion and the anatomical site of that lesion, using the same categories applied in the primary analysis. The original clinical reports were retained as the source for the primary analyses, and the second reading was used solely to quantify inter-reader agreement.

### 2.5. Endpoints

The primary endpoint was deep PSA response, defined as a PSA nadir < 0.2 ng/mL. Progression-free survival (PFS) was the secondary endpoint and was defined as the time from initiation of systemic therapy to radiological disease progression or death from any cause, whichever occurred first. Patients without an event were censored at the date of last follow-up. Death without previously documented radiological progression was considered a PFS event at the date of death. Overall survival (OS) was recorded but was not formally analysed because of the limited number of deaths.

### 2.6. Statistical Analysis

Continuous variables are presented as medians with interquartile ranges (IQRs), and categorical variables as frequencies and percentages. Categorical variables were compared using the chi-square test or Fisher’s exact test, as appropriate. Continuous variables were compared using the Mann–Whitney U test for two groups and the Kruskal–Wallis test for three or more groups. Correlations were assessed using Spearman’s rank correlation coefficient. Trends across prostate-, node-, and bone-dominant groups were evaluated using the ordered dominant-site variable. All statistical tests were two-sided.

Inter-reader agreement for SUVmax was quantified using the two-way random-effects, absolute-agreement, single-measures intraclass correlation coefficient, supplemented by Bland–Altman analysis of the systematic difference and limits of agreement. Agreement for the anatomical site of the dominant lesion was quantified using Cohen’s kappa with bootstrap confidence intervals, both for the full site classification and for the binary contrast of bone-dominant versus all other sites.

Deep PSA response was analysed using binary logistic regression. The association between bone-dominant disease and deep PSA response was evaluated in univariable analysis and sequentially adjusted for potential confounders. A multivariable model including baseline PSA, CHAARTED disease volume, and LDH was additionally fitted. Because disease burden differed according to dominant lesion site, analyses were also performed within strata defined by disease burden.

Progression-free survival was estimated using the Kaplan–Meier method and compared using the log-rank test. Median follow-up was estimated using the reverse Kaplan–Meier method. Hazard ratios (HRs) and 95% confidence intervals (CIs) were estimated using Cox proportional-hazards regression.

Because deep PSA response was determined from PSA measurements obtained during the early treatment period, survival analyses according to response depth were performed using a six-month landmark approach to minimise immortal-time bias. Patients who experienced a PFS event or were censored before the landmark were excluded from the landmark analysis, and survival time was calculated from the landmark onward. Analyses from treatment initiation were additionally performed for comparison with previously published studies.

Because of the small number of PFS events among patients achieving a deep PSA response and the resulting risk of separation, Cox models were fitted using Firth’s penalised partial-likelihood approach. Confidence intervals were derived from the profile penalised likelihood, and *p* values from penalised likelihood-ratio tests. Given the limited number of events, multivariable survival models were restricted to a maximum of two covariates. The proportional-hazards assumption was assessed using scaled Schoenfeld residuals.

To account for multiple testing, the Benjamini–Hochberg procedure was used to control the false discovery rate (FDR). Both nominal *p* values and FDR-adjusted q values are reported, and findings that did not remain statistically significant after FDR correction were considered exploratory.

Statistical analyses were performed using Python version 3.12 (Python Software Foundation, Wilmington, DE, USA; https://www.python.org) with the lifelines (https://lifelines.readthedocs.io), statsmodels (https://www.statsmodels.org), SciPy (https://scipy.org), NumPy (https://numpy.org) and pandas (https://pandas.pydata.org) packages. With 100 evaluable patients and a deep response rate of 51%, the study had 80% power at a two-sided alpha of 0.05 to detect an odds ratio of 0.32 for a dichotomised imaging variable and of approximately 1.8 per standard deviation of log_2_ SUVmax. Analyses were conducted at the patient level.

## 3. Results

### 3.1. Cohort

A total of 105 patients were included in the study. The median age was 71 years (IQR, 67–77), and the median baseline PSA level was 116 ng/mL (IQR, 50–400). Ninety patients presented with de novo metastatic disease. Bone, nodal, and visceral metastases were present in 90, 81, and 22 patients, respectively. According to the CHAARTED criteria, 75 patients had high-volume disease, while 64 met the LATITUDE high-risk criteria. Baseline total testosterone was above the castrate level in all patients, with a median concentration of 425 ng/dL (IQR, 321–555). Following initiation of ADT, all patients achieved castrate testosterone levels (<20 ng/dL).

The median follow-up estimated by the reverse Kaplan–Meier method was 12.7 months (IQR, 7.5–20.0). During follow-up, 17 PFS events occurred, and median PFS was not reached. Evaluable baseline PSMA PET/CT was available in 100 patients, among whom 15 PFS events occurred.

PSA declines of ≥50% and ≥90% from baseline were observed in 103 (98%) and 99 (94%) patients, respectively. A deep PSA response, defined as a nadir <0.2 ng/mL, was achieved by 54 patients (51%). Baseline characteristics according to the anatomical site of the dominant lesion are presented in Table 1. All three on-treatment PSA measurements were available in 101 patients (96%), and the nadir occurred at the six-month measurement in 88. Restricting the analyses to these 101 patients gave essentially unchanged results (bone dominance and deep response: OR 0.23, 95% CI 0.09–0.58).

Inter-reader agreement was excellent for SUVmax (ICC 0.99, 95% CI 0.98–0.99; mean difference −0.63, limits of agreement −5.30 to +4.05) and complete for the anatomical site of the dominant lesion (κ = 1.00; 100/100 concordant).

### 3.2. SUVmax

The median SUVmax of the dominant lesion was 16.9 (IQR, 12.3–26.8; range, 3.1–81.5). SUVmax was not associated with deep PSA response, with median values of 14.8 among patients who achieved a deep response and 18.6 among those who did not (*p* = 0.284). Deep response rates were 52%, 60%, 48%, and 44% across ascending SUVmax quartiles, respectively (*p* = 0.705). Similarly, SUVmax was not associated with PFS across the parameterisations examined, including continuous, logarithmic, and categorical analyses (Table 2, Figure 2).

Correlations between SUVmax and baseline PSA (ρ = 0.07) and LDH (ρ = 0.16) were weak. SUVmax did not differ according to CHAARTED disease volume. Although SUVmax was higher in patients with LATITUDE high-risk disease than in those with low-risk disease (median, 21.1 vs. 13.7; *p* = 0.022), this association did not remain statistically significant after correction for multiple testing. Analysed as a continuous variable, SUVmax was not associated with deep PSA response (OR 0.82 per standard deviation of log_2_ SUVmax; 95% CI 0.55–1.22).

### 3.3. Site of the Dominant Lesion

The dominant lesion was located in the prostate in 39 patients, bone in 34, a lymph node in 25, and a visceral or other site in 2. Given the small number of patients with visceral or other dominant lesions, site-based comparisons focused on prostate-, node-, and bone-dominant disease.

Deep PSA response showed an ordered gradient across these three groups. A PSA nadir < 0.2 ng/mL was achieved in 28 of 39 patients with prostate-dominant disease (72%), 13 of 25 with node-dominant disease (52%), and 10 of 34 with bone-dominant disease (29%; overall *p* = 0.001; *p* for trend <0.001). A similar gradient was observed for PSA nadir as a continuous measure, with median values of 0.08, 0.19, and 0.50 ng/mL in the prostate-, node-, and bone-dominant groups, respectively (*p* = 0.008). In contrast, SUVmax did not differ across these groups (median, 14.7, 17.5, and 21.1, respectively; *p* = 0.308). These findings are summarised in Table 3 and illustrated in Figure 3 and Figure 4.

### 3.4. Bone Dominance, Bone Involvement and Disease Burden

Among the 85 patients with bone metastases and evaluable baseline PET/CT, deep PSA response was achieved in 10 of 34 patients with bone-dominant disease (29%) compared with 29 of 51 patients whose dominant lesion was located elsewhere (57%; *p* = 0.016). Patients with bone-dominant disease were more heavily burdened at baseline, with high-volume disease by CHAARTED criteria in 94% versus 61%, LATITUDE high-risk disease in 85% versus 50%, higher LDH and lower haemoglobin (Table 1). Tumour grade did not differ between the groups.

The unadjusted odds ratio for deep response was 0.25 (95% CI 0.10–0.62). Across eleven models adding log SUVmax, CHAARTED volume, LDH, baseline PSA, ISUP grade, number of involved metastatic sites, de novo presentation, prior local therapy, ARPI agent, presence of bone metastases, and a three-covariate combination, the estimate ranged between 0.22 and 0.33, with all confidence intervals excluding unity. Adjustment for the presence of bone metastases alone yielded an odds ratio of 0.32 (95% CI 0.13–0.80). Within strata defined by disease burden, a deep response was reached by 8 of 32 bone-dominant patients (25%) and 23 of 40 others (57%) among the 72 patients with high-volume disease (*p* = 0.008), and by 25% and 54%, respectively, among the 67 patients with both bone metastases and high-volume disease (*p* = 0.024). All models and strata are shown in Table 4.

In a model adjusting simultaneously for LDH, haemoglobin and the number of involved sites, bone dominance remained associated with deep PSA response, although the estimate was attenuated (OR 0.35; 95% CI 0.13–0.95; *p* = 0.040). Neither LDH nor haemoglobin contributed materially to this model (OR 1.00 per U/L and 1.06 per g/dL, respectively). Among the 75 patients with two or more involved sites, in whom metastatic extent was comparable, deep PSA response was achieved in 6 of 30 patients with bone-dominant disease (20%) compared with 23 of 45 others (51%; *p* = 0.008). When bone dominance and CHAARTED volume were included in the same model, bone dominance remained associated with deep PSA response, whereas CHAARTED volume was not (OR, 0.44; 95% CI, 0.16–1.21; *p* = 0.111). Adding lesion site to a model containing CHAARTED volume improved model fit (likelihood-ratio *p* = 0.016), whereas adding CHAARTED volume to a model already containing lesion site did not (*p* = 0.105). Adding lesion site to a model containing CHAARTED volume lowered the AIC from 135.9 to 132.2 and increased the optimism-corrected C-statistic from 0.613 to 0.668 (increment 0.066; bootstrap 95% CI 0.019–0.148).

### 3.5. Depth of PSA Response and Progression

Thirteen patients were excluded from the six-month landmark analysis (four with an event and nine censored before the landmark), leaving 92 patients with 13 events. Excluded and included patients did not differ significantly across fourteen baseline variables, including bone dominance and CHAARTED volume (Table S1), although this comparison has limited power. In the six-month landmark analysis, progression occurred in 1 of 49 patients who achieved a deep PSA response and in 12 of 43 patients who did not. Deep PSA response was associated with longer PFS (HR, 0.10; 95% CI, 0.01–0.42), with estimated 12-month PFS rates of 97% (95% CI, 80–100) and 71% (95% CI, 52–83), respectively (Figure 5). Estimates beyond 12 months are based on few patients remaining at risk (n = 2 and n = 3 at 24 months) and are not reported. An early (three-month) deep PSA response showed a similar association that did not survive correction for multiple testing (Figure S1).

In exploratory analyses of clinical and laboratory variables, LDH above the cohort median was associated with shorter PFS after adjustment for deep PSA response (HR, 3.35; 95% CI, 1.13–13.03); the full set of laboratory markers is shown in Table S2 and Figure S2.

Bone-dominant disease was not associated with PFS (HR, 1.40; 95% CI, 0.47–4.05). When bone dominance and deep PSA response were included in the same model, the HR for bone dominance was attenuated (HR, 0.78; 95% CI, 0.27–2.21), whereas the association between deep PSA response and PFS remained of similar magnitude. An exploratory decomposition of these associations is presented in Table S3. Survival analyses are presented in Table 5.

### 3.6. Model Assumptions, Multiplicity and Selection

No evidence of violation of the proportional-hazards assumption was observed in the fitted Cox models (lowest *p* = 0.14). After Benjamini–Hochberg correction for multiple testing, the principal findings remained statistically significant (Table S4), including the ordered association between dominant lesion site and deep PSA response (q = 0.008), the association between deep PSA response and PFS in the landmark analysis (q = 0.010), the overall association between dominant lesion site and deep PSA response (q = 0.014), and the association between bone-dominant disease and deep PSA response (q = 0.016). Excluded patients did not differ from the analysed cohort in SUVmax, site of the dominant lesion or ARPI agent, but were less likely to have undergone follow-up PET/CT (28% versus 48%, *p* = 0.025; Table S5).

## 4. Discussion

In this cohort of patients with mHSPC treated with contemporary ARPI-based therapy, conventional PSA responses were nearly universal, with PSA50 and PSA90 achieved by 98% and 94% of patients, respectively. In contrast, a PSA nadir <0.2 ng/mL was achieved by approximately half of the cohort, providing greater discrimination in the depth of treatment response. Against this background, two features derived from the most PSMA-avid lesion on baseline PET/CT provided different information. SUVmax was not associated with either the depth of PSA response or PFS across the parameterisations examined, within the limits of what the study could detect. In contrast, the anatomical site of the dominant lesion was associated with the depth of PSA response, with an ordered gradient from prostate- to node- to bone-dominant disease that was evident both in the proportion achieving a deep response and in the PSA nadir itself.

The absence of an association between SUVmax and outcome warrants comparison with previous reports. PSMA uptake metrics have demonstrated prognostic relevance in castration-resistant disease and in patients undergoing PSMA-targeted radioligand therapy [17], whereas evidence in the hormone-sensitive setting remains more limited. In 82 men with metachronous mHSPC, SUVmax was reported to predict PSA response to ADT combined with an ARPI, although that cohort used PSA50 rather than the nadir as its endpoint and was restricted to metachronous disease [22]. In another cohort of 165 men followed for a median of 44 months, SUVmax > 17.16 was associated with shorter radiographic PFS [23]. Notably, patients with higher SUVmax in that study also had poorer performance status, more frequent de novo and high-volume disease, and higher alkaline phosphatase levels, suggesting that the observed association may have partly reflected differences in disease burden. Moreover, the reported threshold was data-derived and provided relatively modest discrimination, with a concordance index of 0.60.

Several factors may explain the absence of an association between baseline SUVmax and treatment response in our cohort. PSMA expression is influenced by androgen receptor signalling, and all baseline scans in this study were obtained before initiation of androgen suppression. Baseline uptake may therefore reflect a biological state that is subsequently altered by ADT and ARPI therapy rather than a stable tumour characteristic maintained during treatment. In addition, SUVmax is inherently sensitive to technical and acquisition-related factors, particularly when derived retrospectively from routine clinical reports. These considerations may limit the prognostic information provided by an isolated baseline SUVmax in the hormone-sensitive setting.

The association with dominant lesion site was more pronounced. Deep PSA response was achieved by 72%, 52%, and 29% of patients with prostate-, node-, and bone-dominant disease, respectively. This ordered gradient was also evident in the PSA nadir itself, supporting the observation across both categorical and continuous measures of response. Bone-dominant disease was associated with substantially lower odds of achieving a deep PSA response (unadjusted OR, 0.25), and the magnitude of this association remained consistent across sequentially adjusted models.

A similar anatomical gradient has been reported in survival analyses. In a series of 451 men with mHSPC stratified according to the distribution of metastatic disease, median overall survival decreased from 95 months in patients with nodal disease to 64 and 50 months in those with low- and high-volume bone disease, respectively, and to 46 months in those with visceral involvement [24]. Although metastatic distribution and the site of the dominant lesion represent different constructs, this anatomical gradient is directionally consistent with the pattern observed in our cohort. Our findings extend this observation to treatment response, suggesting that anatomical disease distribution may also be related to the depth of PSA suppression achieved with contemporary systemic therapy. Whether differences in response depth contribute to the established prognostic effect of metastatic distribution requires further investigation.

A closer parallel comes from PSMA PET imaging. In a recent analysis of baseline PSMA PET in castration-resistant prostate cancer, lesion location independently predicted subsequent lesion progression, with lower odds of progression for prostatic than for osseous lesions (OR, 0.23), alongside SUVmean (OR, 1.72 per doubling), whereas radiomic features were not independently informative [25]. That study was conducted at the individual-lesion level in castration-resistant disease and used lesion progression as the endpoint, whereas our analysis was performed at the patient level in hormone-sensitive disease using deep PSA response as the primary outcome. The estimates are therefore not directly comparable. Nevertheless, the observation that anatomical location carries information across different disease settings, units of analysis, and endpoints supports further investigation of lesion site as an imaging feature distinct from uptake intensity. Anatomical heterogeneity may also exist within bone itself, as skull involvement has been associated with shorter progression-free and overall survival than involvement of other osseous sites [26].

Not all reports agree, and these discrepancies may help clarify which aspects of metastatic distribution carry prognostic information. In a national registry of newly diagnosed metastatic prostate cancer, concomitant non-regional nodal and bone involvement was associated with poorer prognosis than bone-only disease among patients with low-volume disease [27]. Such analyses describe which anatomical sites are involved, rather than the location of the most PSMA-avid lesion. Our findings suggest that these features may provide distinct information. Bone metastases were present in the majority of our cohort, whereas identification of bone as the site of the dominant lesion was associated with a lower probability of deep PSA response. Importantly, this association remained after adjustment for the presence of bone metastases itself.

A biological basis for an association with lesion site is plausible. Bone is not simply a passive destination for metastatic prostate cancer but provides a microenvironment capable of supporting tumour-cell survival. Under androgen receptor-directed therapy, osteoblasts in the bone microenvironment secrete factors that promote tumour cell proliferation and anti-androgen resistance [20], while disseminated prostate cancer cells can directly compete with haematopoietic stem cells for occupancy of the bone marrow niche [21]. These microenvironmental interactions provide a potential biological rationale for the lower probability of deep PSA suppression observed when the most PSMA-avid lesion was located in bone, although the present study cannot establish the underlying mechanism.

This distinction may also help contextualise the heterogeneous prognostic associations reported for metastatic site in previous studies, which have generally classified patients according to the presence or absence of metastases at specific anatomical locations rather than the site of the most PSMA-avid lesion. Osseous metastases themselves are biologically heterogeneous, encompassing molecular phenotypes that differ in proliferation, androgen receptor signalling and prognosis [28]. Accordingly, the prognostic implications of bone involvement may not be uniform across patients.

An immediate question is whether the information provided by dominant lesion site is already captured by established disease-burden classifications, since the CHAARTED definition incorporates the extent of osseous disease and the LATITUDE criteria include the number of bone lesions [4,6]. Although these classifications are well-established prognostic tools, there remains interest in refining risk stratification with additional markers [29]. Our findings suggest that disease volume and dominant lesion site capture different aspects of metastatic disease. Adding dominant lesion site to a model containing CHAARTED volume improved model fit (*p* = 0.016) and discrimination (optimism-corrected C-statistic 0.613 to 0.668), whereas adding CHAARTED volume to a model already containing lesion site did not (*p* = 0.105). The association was also observed within burden-defined strata. Among patients with high-volume disease, deep PSA response was achieved in 25% of those with bone-dominant disease compared with 57% of those whose dominant lesion was located elsewhere (*p* = 0.008), with similar patterns observed in other burden-defined subgroups. Tumour grade did not differ according to dominant lesion site, suggesting that bone dominance was not simply a surrogate for conventional histological grade. Disease-volume classifications quantify the extent of metastatic burden, whereas dominant lesion site identifies the anatomical location of the most PSMA-avid lesion. Patients within the same CHAARTED category may therefore differ in this imaging feature, and our findings suggest that this distinction may provide additional information regarding the depth of treatment response.

LDH was the only clinical variable associated with PFS after adjustment for deep PSA response, although this analysis was exploratory rather than prespecified. This finding is consistent with previous evidence supporting the prognostic relevance of LDH in metastatic prostate cancer. A meta-analysis of 59 studies including 14,851 patients found elevated LDH to be associated with worse overall survival (pooled HR, 2.07; 95% CI, 1.75–2.44), with a similar association in the hormone-sensitive subgroup (pooled HR, 2.25; 95% CI, 1.78–2.84), whereas the pooled association with PFS was substantially weaker (HR, 1.08; 95% CI, 1.01–1.16) [30]. Broader analyses across solid tumours have likewise identified prostate cancer among the malignancies in which LDH carries prognostic information [31]. As a marker related to tumour burden and metabolic activity, LDH may capture aspects of disease biology that are not reflected by PSA response alone.

Taken together, our findings distinguish between imaging features associated with treatment response and those associated with subsequent progression. Dominant lesion site was associated with the depth of PSA response but was not directly associated with PFS, whereas deep PSA response showed a strong association with subsequent PFS in the landmark analysis. The prognostic relevance of PSA nadir is consistent with long-term evidence from CHAARTED, in which depth of PSA response was associated with long-term outcomes [12]. These findings raise the hypothesis that anatomical characteristics of metastatic disease may be related to the depth of treatment response, while the achieved response itself may be more closely related to subsequent disease control. Given the limited number of progression events and the observational design, this proposed relationship requires validation in larger prospective cohorts. The mediation analysis is exploratory and based on 15 events.

An alternative interpretation should also be considered. PSA nadir is a surrogate of disease control and may not reflect residual viable tumour equally across anatomical sites. If bone lesions differ from prostatic or nodal lesions in PSA production relative to viable tumour burden, or if patients with bone-dominant disease harbour a greater burden not fully captured by conventional volume classifications, differences in PSA nadir could arise without corresponding differences in biological treatment response. Several observations provide context for this possibility. Molecular profiling of prostate cancer bone metastases has identified biologically aggressive phenotypes characterised by high proliferation and relatively low PSA immunoreactivity, and these phenotypes are associated with poorer prognosis [28], suggesting that differences in PSA production alone may not readily explain the higher nadirs observed in patients with bone-dominant disease. Moreover, the association between bone dominance and deep PSA response remained consistent after accounting for disease-volume classification and the number of involved metastatic sites. Nevertheless, residual confounding by disease burden cannot be excluded. The proposed biological rationale should therefore be regarded as a hypothesis rather than an explanation established by these data, since the design cannot distinguish a genuine microenvironmental effect from a difference in PSA production. PSA nadir is a surrogate endpoint, and resolving this ambiguity requires PSA-independent measures of response. Circulating tumour DNA is one such measure. It is not directly regulated by androgen receptor signalling and therefore captures different elements of tumour activity, and in metastatic hormone-sensitive disease, persistent ctDNA detection during first-line intensified therapy predicts survival earlier than PSA kinetics [32]. Its shedding also varies by the anatomical distribution of disease, being lowest in nodal-only disease and increasing with bone lesion number [33], so it would complement rather than replace anatomically resolved measures. Neither was available in the present study. PSMA PET-derived volumetric approaches are increasingly being investigated as imaging-based measures of disease burden [34], while multimodal models integrating digital histopathology and clinical variables have also demonstrated prognostic potential in mHSPC cohorts derived from CHAARTED and STAMPEDE [35,36].

Our analysis focused on SUVmax because it is readily available from routine clinical PET/CT reports. Volumetric PSMA PET metrics capture a broader representation of total disease burden and have demonstrated prognostic value in advanced prostate cancer [16]. Whether the anatomical site of the dominant lesion provides information beyond such volumetric metrics, or is largely captured by them, cannot be determined from the present data. Nevertheless, dominant lesion site has the practical advantage of being immediately available from routine staging PSMA PET/CT without additional segmentation or post-processing. If validated prospectively, this simple imaging feature may complement established disease-burden classifications in identifying patients less likely to achieve deep PSA suppression and may contribute to risk stratification and subsequent monitoring.

### Limitations

This study has several limitations. First, its retrospective, single-centre design and relatively small sample size limit generalisability. Only 17 PFS events occurred during a median follow-up of 12.7 months, which is short relative to the progression-free survival reported with contemporary ARPI-based therapy; median PFS was therefore not reached as a consequence of limited follow-up rather than as evidence of durable disease control. Only 14 patients were followed beyond 24 months, and survival estimates at later time points were based on progressively smaller numbers of patients at risk and overall survival could not be formally analysed. With 100 evaluable patients, the study had 80% power to detect an odds ratio of 0.32 for a dichotomised imaging variable. The observed estimate for SUVmax (OR 0.82, 95% CI 0.55–1.22) excludes only associations stronger than this, and the null result should be read accordingly.

Second, imaging variables were derived from routine clinical PET/CT reports rather than from a standardised central imaging review. A blinded second reading of all baseline scans showed excellent agreement for SUVmax and complete agreement for the anatomical site of the dominant lesion, but both readers were nuclear medicine physicians from the same institution with comparable experience, and the agreement observed may therefore not reflect reproducibility across centres or across readers of differing experience. The primary analyses were based on the original clinical reports rather than on the second reading. The negative findings for SUVmax should also be interpreted in the context of a routinely reported single-lesion metric rather than as evidence against the potential prognostic relevance of PSMA uptake more broadly. Volumetric PSMA PET metrics could not be derived retrospectively and were not evaluated. Follow-up PET/CT was available only in a subset of patients, and quantitative SUVmax measurements were not consistently recorded; consequently, longitudinal metabolic response could not be assessed.

Third, residual selection bias and confounding cannot be excluded. Patients excluded because of insufficient data or follow-up could not be fully characterised across all baseline clinical and laboratory variables. Treatment allocation was not randomised and reflected routine clinical decision-making; therefore, despite the absence of marked differences in response according to ARPI agent, unmeasured confounding by treatment selection remains possible.

Finally, multiple statistical comparisons were performed in the context of a relatively small cohort. False-discovery-rate correction was applied to reduce the risk of false-positive findings, and results not remaining statistically significant after correction were treated as exploratory. Nevertheless, the findings should be considered hypothesis-generating and require external validation in larger prospective cohorts with longer follow-up and standardised imaging assessment before clinical application. Transcriptomic approaches integrating immune microenvironment profiling with drug resistance signatures have similarly been proposed for prognostication in prostate cancer, although these have been derived largely from primary tumour datasets [37].

## 5. Conclusions

In ARPI-treated mHSPC, the anatomical site of the dominant PSMA PET/CT lesion was associated with the depth of PSA response, whereas its SUVmax was not. Bone-dominant disease was associated with a substantially lower probability of achieving a PSA nadir <0.2 ng/mL, independent of the presence of bone metastases, and this association remained consistent across adjusted and disease-burden-stratified analyses. Deep PSA response was associated with longer PFS, whereas bone dominance itself was not. These findings suggest that the anatomical site and uptake intensity of the dominant lesion may provide distinct information and warrant prospective validation in larger cohorts with standardised imaging assessment.

## Figures and Tables

**Figure 1:**
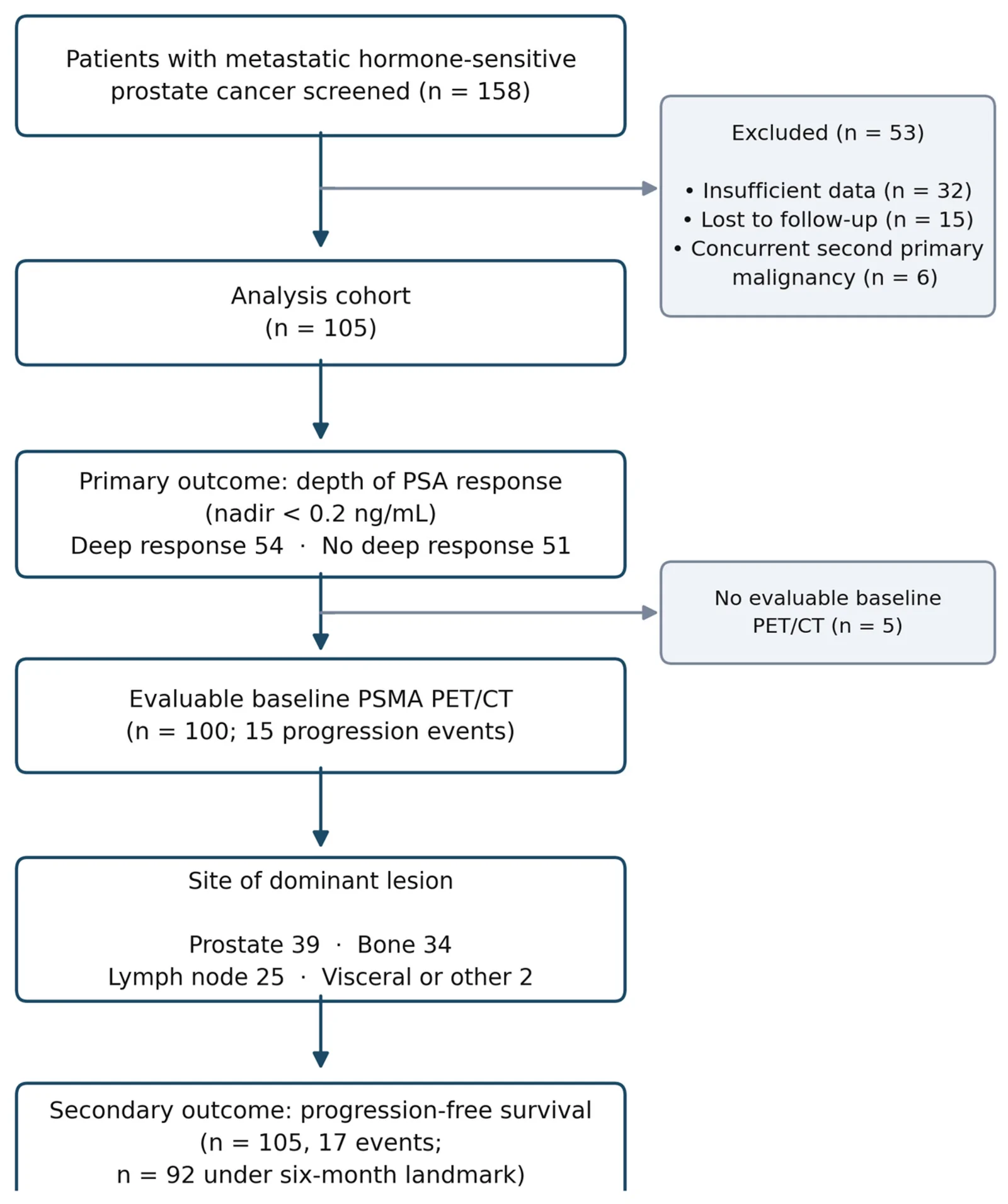
Flow diagram of patient selection.

**Figure 2:**
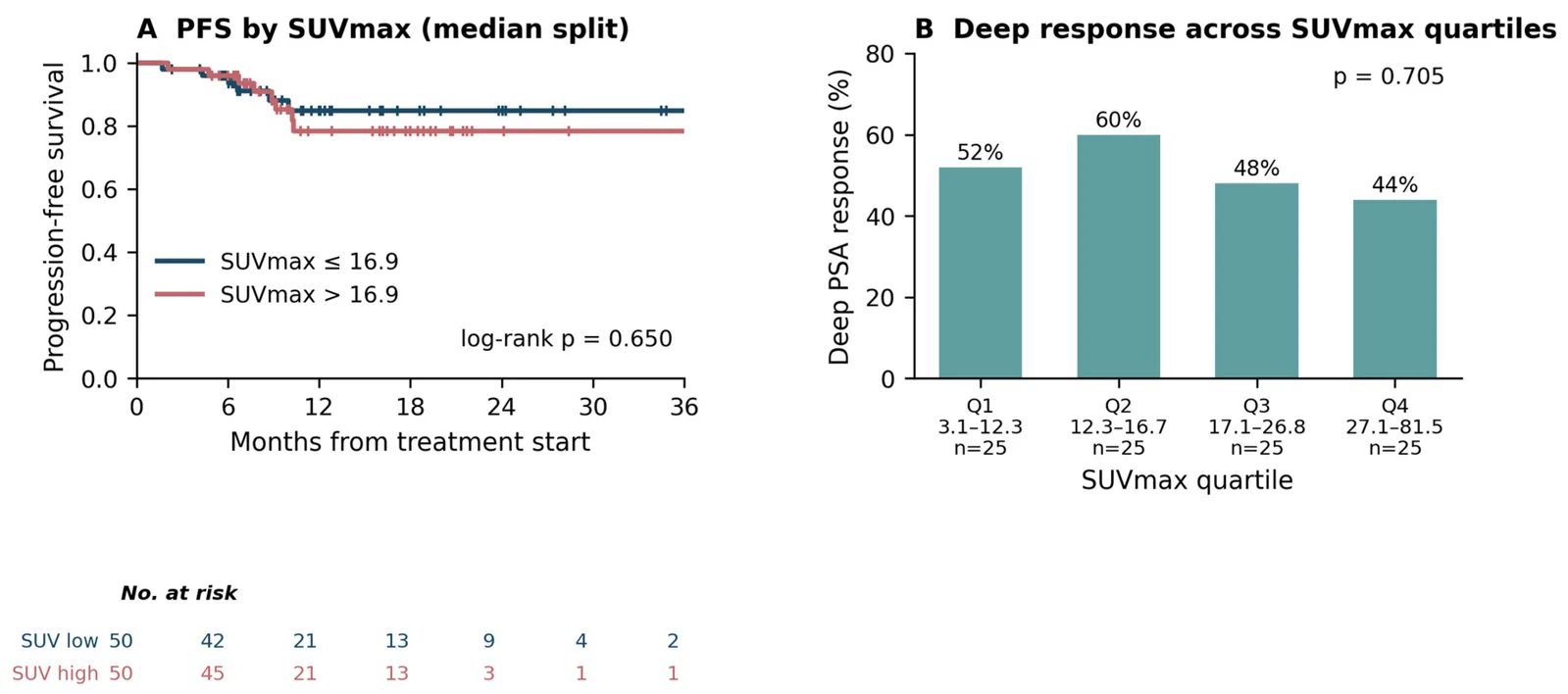
SUVmax and outcome. (**A**) Progression-free survival by baseline SUVmax dichotomised at the cohort median; (**B**) deep PSA response across SUVmax quartiles, with the SUVmax range and number of patients shown beneath each bar.

**Figure 3:**
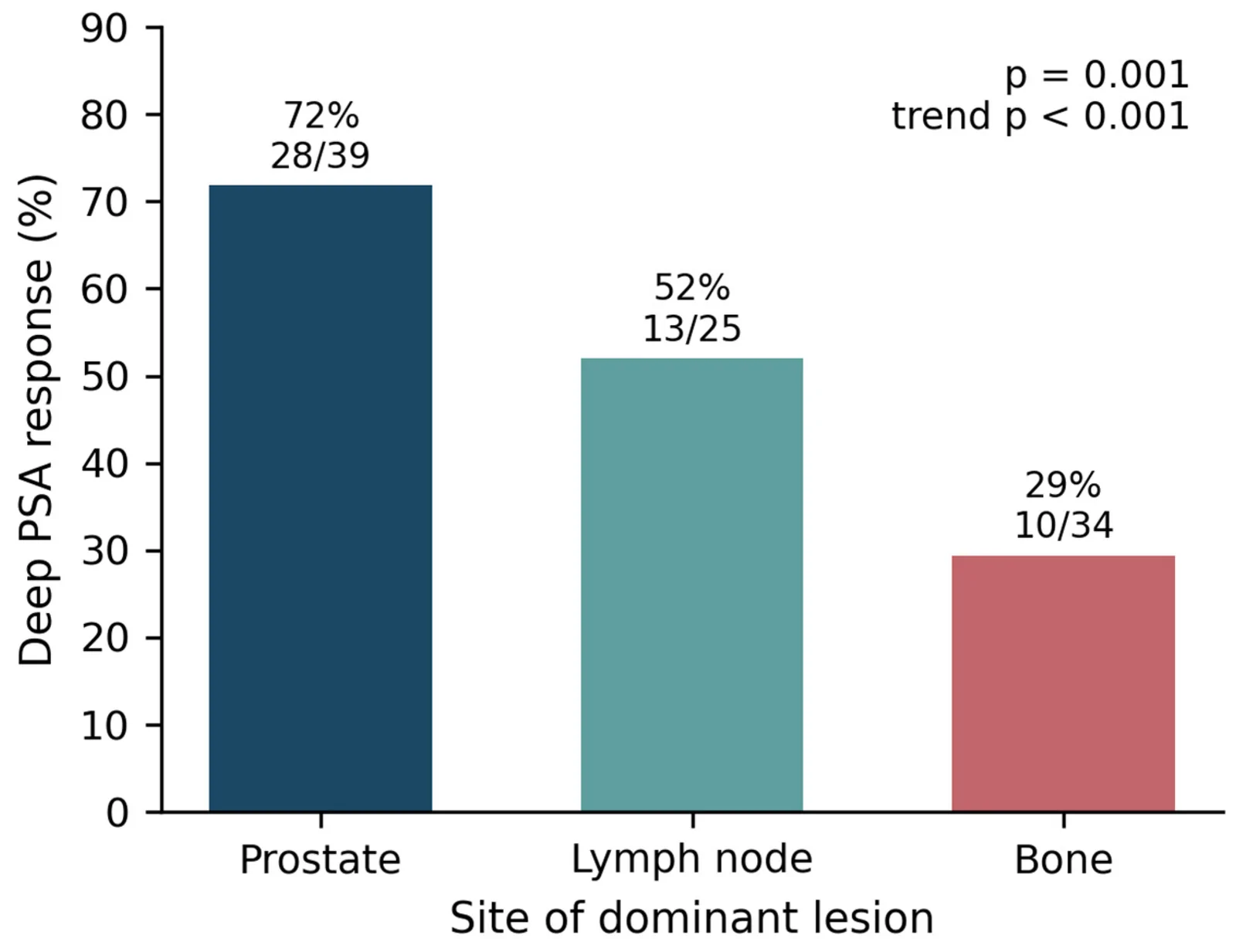
Deep PSA response by the site of the dominant lesion at baseline.

**Figure 4:**
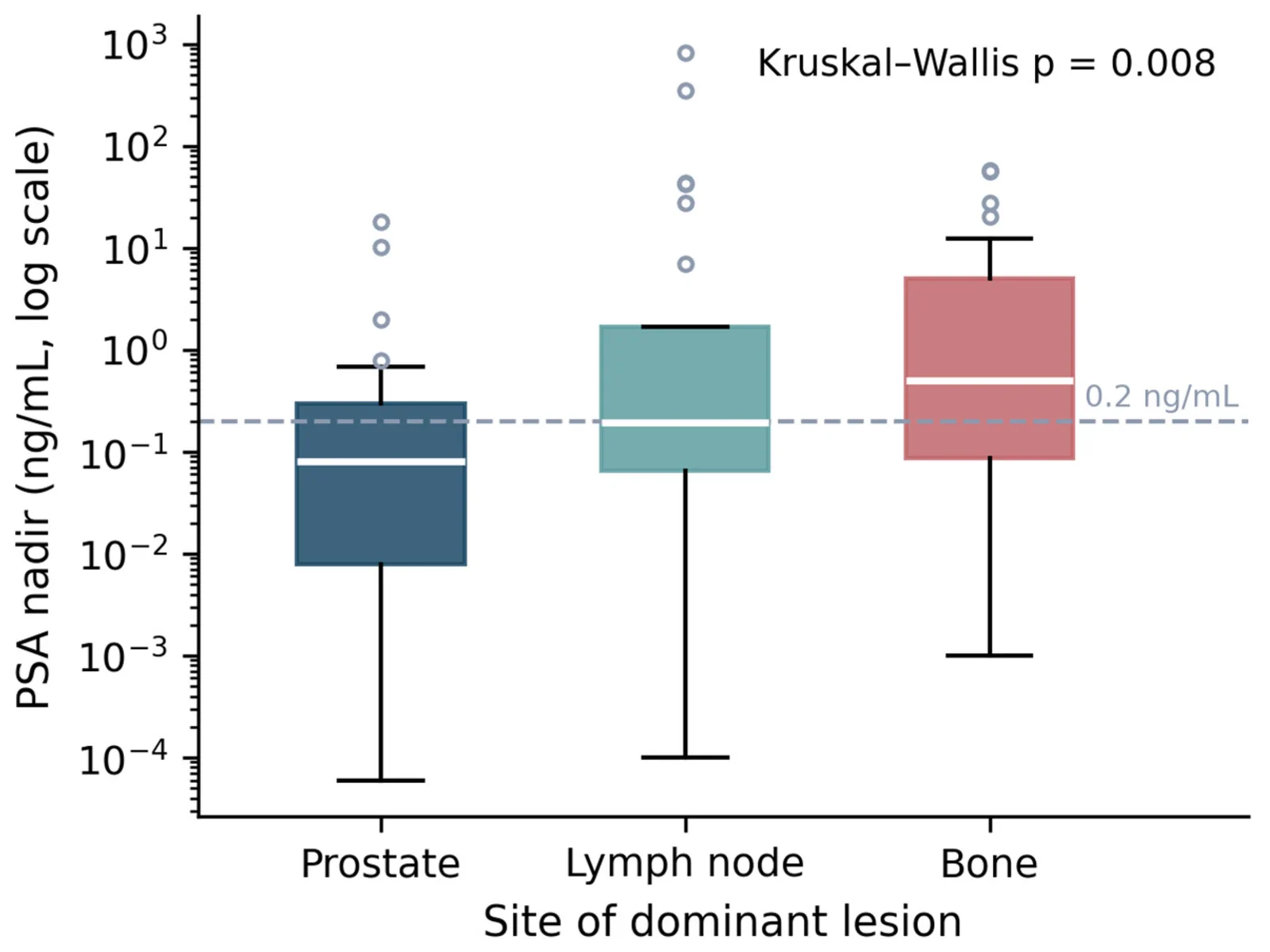
PSA nadir by the site of the dominant lesion. The dashed line marks the 0.2 ng/mL threshold; the y-axis is logarithmic. Circles denote outliers.

**Figure 5:**
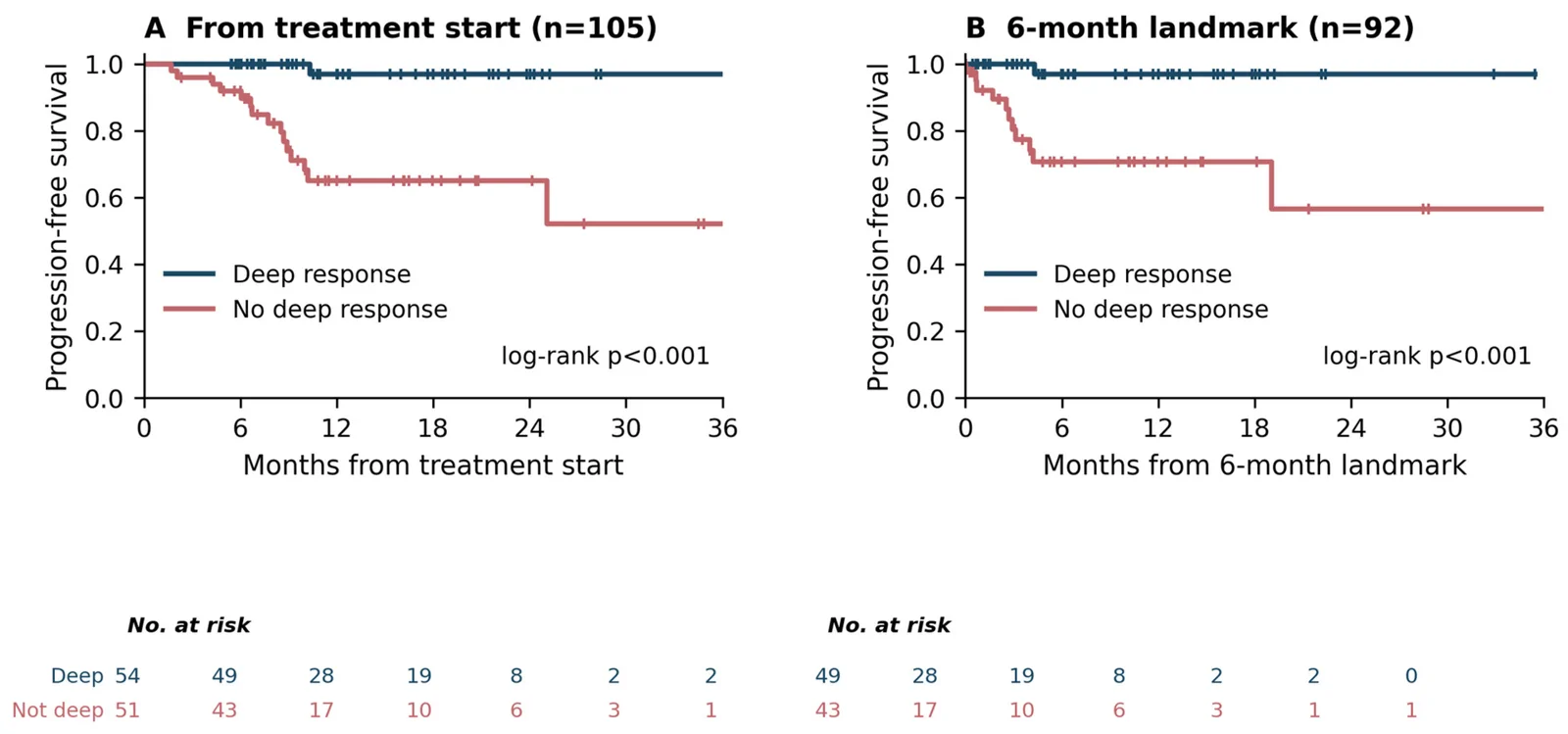
Progression-free survival by depth of PSA response. (**A**) From treatment start; (**B**) under a six-month landmark, which removes the immortal-time advantage inherent in defining response depth from the first six months of follow-up. Censored observations are marked with a cross; numbers at risk are shown beneath each panel.

**Table 1:** Baseline characteristics according to the site of the dominant PET lesion (n = 100).

Characteristic	Bone-Dominant (n = 34)	Other Dominance (n = 66)	*p*
Age, median (IQR), years	74.5 (70.0–79.8)	70.5 (65.2–75.8)	0.008
ECOG performance status 0–1, n (%)	31 (91%)	64 (97%)	0.333
Baseline PSA, median (IQR), ng/mL	164.5 (90.3–547.8)	98.5 (49.0–320.0)	0.108
ISUP grade group ≥ 4, n (%)	26 (76%)	53 (80%)	0.796
De novo metastatic presentation, n (%)	27 (79%)	59 (89%)	0.225
Prior local therapy, n (%)	9 (26%)	19 (29%)	1.000
Bone metastases, n (%)	34 (100%)	51 (77%)	0.002
Nodal metastases, n (%)	25 (74%)	55 (83%)	0.294
Visceral metastases, n (%)	9 (26%)	12 (18%)	0.437
Involved sites, median (IQR)	2 (2–2)	2 (1–2)	0.073
CHAARTED high volume, n (%)	32 (94%)	40 (61%)	<0.001
LATITUDE high risk, n (%)	29 (85%)	33 (50%)	0.001
SUVmax, median (IQR)	21.1 (12.7–34.4)	15.8 (12.3–26.4)	0.227
LDH, median (IQR), U/L	223 (174–305)	186 (163–216)	0.010
Albumin, median (IQR), g/L	41.0 (37.6–43.8)	43.0 (40.0–45.0)	0.108
Haemoglobin, median (IQR), g/dL	12.1 (10.3–13.6)	13.8 (11.9–14.8)	0.003
Enzalutamide, n (%)	13 (38%)	29 (44%)	0.671
Abiraterone, n (%)	14 (41%)	21 (32%)	0.382
Apalutamide, n (%)	4 (12%)	13 (20%)	0.406
Triplet regimen, n (%)	3 (9%)	3 (5%)	0.406

IQR, interquartile range; ECOG, Eastern Cooperative Oncology Group; ISUP, International Society of Urological Pathology; LDH, lactate dehydrogenase. Bone dominance denotes a dominant (highest-SUVmax) lesion lying in bone. Continuous variables were compared with the Mann–Whitney U test and proportions with Fisher’s exact test.

**Table 2:** SUVmax of the dominant lesion across all parameterisations examined (n = 100).

Parameterisation	Effect Estimate	95% CI	*p*
Progression-free survival			
Continuous, per unit	HR 1.00	0.97–1.03	0.931
Per doubling (log_2_ scale)	HR 1.24	0.77–2.02	0.375
Dichotomised at median (>16.9)	HR 1.28	0.44–3.68	0.651
Dichotomised at upper quartile (>26.8)	HR 1.11	0.35–3.54	0.861
Tertiles (2/6/7 events)	–	–	0.162
Cut-point sweep, SUVmax 8–35 (28 thresholds)	–	–	min 0.090
Depth of PSA response			
SUVmax, deep vs. no deep response	14.8 vs. 18.6	–	0.284
Deep response, low vs. high SUVmax	56% vs. 46%	–	0.424
Deep response across ascending quartiles	52/60/48/44%	–	0.705
Associations with other baseline markers			
SUVmax vs. baseline PSA	rho 0.07	–	0.479
SUVmax vs. LDH	rho 0.16	–	0.101
SUVmax vs. CHAARTED high volume	17.2 vs. 14.7	–	0.631
SUVmax vs. LATITUDE high risk	21.1 vs. 13.7	–	0.022

HR, hazard ratio; CI, confidence interval; LDH, lactate dehydrogenase. Cox models were fitted with Firth’s penalised partial likelihood. The cut-point sweep is reported to document the absence of an identifiable threshold; its minimum *p* value is uncorrected for the 28 implicit comparisons and should not be interpreted as a test. The association with LATITUDE high-risk disease did not survive false-discovery-rate correction (q = 0.055). Quartile analyses divide the cohort into groups of 25 patients, in which power to detect a difference is limited.

**Table 3:** Depth of PSA response according to the site of the dominant lesion.

Site of Dominant Lesion	n	Deep Response, n (%)	PSA Nadir, Median (ng/mL)
Prostate	39	28 (72%)	0.08
Lymph node	25	13 (52%)	0.19
Bone	34	10 (29%)	0.50
Visceral or other	2	–	–
Chi-square across three sites		*p* = 0.001	
Ordered trend (prostate to node to bone)		*p* < 0.001	
Kruskal–Wallis for PSA nadir			*p* = 0.008
SUVmax across sites (14.7/17.5/21.1)			*p* = 0.308

A deep response denotes a PSA nadir below 0.2 ng/mL. Formal testing was restricted to the three sites with adequate numbers; the two patients with visceral or other dominance are shown for completeness. The trend was tested by Spearman rank correlation against the ordered site sequence.

**Table 4:** Bone dominance and deep PSA response: adjusted odds ratios and analyses within strata defined by disease burden (n = 100).

Model or Stratum	Odds Ratio/Comparison	95% CI	*p*
Unadjusted	OR 0.25	0.10–0.62	0.003
Adjusted, one covariate at a time			
+log SUVmax	OR 0.26	0.11–0.64	0.003
+CHAARTED volume	OR 0.33	0.13–0.83	0.019
+LDH	OR 0.27	0.11–0.65	0.004
+log baseline PSA	OR 0.26	0.10–0.67	0.005
+ISUP grade ≥ 4	OR 0.22	0.08–0.56	0.002
+number of involved sites	OR 0.29	0.11–0.74	0.010
+de novo metastatic presentation	OR 0.22	0.09–0.56	0.002
+prior local therapy	OR 0.26	0.10–0.62	0.003
+ARPI agent	OR 0.27	0.11–0.66	0.004
+presence of bone metastases	OR 0.32	0.13–0.80	0.014
Multivariable models			
+PSA, CHAARTED volume and LDH	OR 0.27	0.10–0.74	0.011
+LDH, haemoglobin and number of involved sites	OR 0.35	0.13–0.95	0.040
Strata defined by disease burden (unadjusted)			
Bone metastases present (n = 85)	10/34 (29%) vs. 29/51 (57%)	–	0.016
CHAARTED high volume (n = 72)	8/32 (25%) vs. 23/40 (57%)	–	0.008
LATITUDE high risk (n = 62)	7/29 (24%) vs. 17/33 (52%)	–	0.038
Bone metastases and high volume (n = 67)	8/32 (25%) vs. 19/35 (54%)	–	0.024
Two or more involved sites (n = 75)	6/30 (20%) vs. 23/45 (51%)	–	0.008

OR, odds ratio; LDH, lactate dehydrogenase. Odds ratios below unity indicate a lower probability of achieving a PSA nadir below 0.2 ng/mL in bone-dominant disease. Each model in the first section adds a single covariate to the unadjusted logistic model. In the four-covariate model, the number of involved sites was dichotomised as one versus two or more, since the relationship with deep response was not linear across categories; modelling it as a continuous variable gave OR 0.31 (95% CI 0.12–0.82). Neither LDH (OR 1.00 per U/L) nor haemoglobin (OR 1.06 per g/dL) contributed materially to that model. Stratified comparisons were tested with Fisher’s exact test.

**Table 5:** Progression-free survival analyses.

Comparison	Events	Hazard Ratio (95% CI)	*p*
Deep PSA response, from treatment start	1/54 vs. 16/51	0.08 (0.01–0.31)	<0.001
Deep PSA response, six-month landmark	1/49 vs. 12/43	0.10 (0.01–0.42)	0.001
LDH above median, from treatment start	14/52 vs. 3/53	4.19 (1.43–16.27)	0.008
LDH above median, six-month landmark	–	–	0.013
Bone dominance, unadjusted	7/34 vs. 8/66	1.40 (0.47–4.05)	0.471
Bone dominance, adjusted for response depth	–	0.78 (0.27–2.21)	0.645
Deep response and LDH, joint model			
Deep PSA response	–	0.09 (0.01–0.36)	<0.001
LDH above median	–	3.35 (1.13–13.03)	0.028
Deep response and LDH, six-month landmark			
Deep PSA response	–	0.12 (0.01–0.49)	0.002
LDH above median	–	3.82 (1.09–19.91)	0.035

LDH, lactate dehydrogenase; CI, confidence interval. All models were fitted with Firth’s penalised partial likelihood, with profile-likelihood confidence intervals, because a single event among patients with a deep response makes Wald-based intervals unreliable. Analyses of response depth and LDH include all 105 patients (17 events); those involving bone dominance are restricted to the 100 patients with evaluable baseline PET (15 events). The proportional-hazards assumption was satisfied in every model (lowest *p* = 0.14). The LDH finding was exploratory.

## Data Availability

The data presented in this study are available on reasonable request from the corresponding author. The data are not publicly available due to patient privacy restrictions.

## Supplementary Materials

The following supporting information can be downloaded at: https://www.mdpi.com/article/10.3390/biomedicines14102160/s1. Table S1: Baseline characteristics of patients excluded at the six-month landmark compared with those included in the landmark analysis; Table S2: Baseline laboratory markers and depth of PSA response; Table S3: Exploratory decomposition of the associations between bone dominance, depth of PSA response and progression-free survival; Table S4: Complete register of all 38 hypothesis tests performed, with unadjusted *p* values and Benjamini–Hochberg adjusted q values; Table S5: Comparison of excluded and analysed patients across baseline variables available in both groups; Figure S1: Progression-free survival by early (three-month) deep PSA response under a three-month landmark; Figure S2: Baseline albumin and haemoglobin by depth of PSA response.

## Abbreviations

The following abbreviations are used in this manuscript:

^68^Ga-PSMA-11	Gallium-68 prostate-specific membrane antigen-11
ADT	Androgen deprivation therapy
ARPI	Androgen receptor pathway inhibitor
CI	Confidence interval
CT	Computed tomography
ECOG	Eastern Cooperative Oncology Group
FDR	False discovery rate
HR	Hazard ratio
IQR	Interquartile range
ISUP	International Society of Urological Pathology
LDH	Lactate dehydrogenase
mHSPC	Metastatic hormone-sensitive prostate cancer
OR	Odds ratio
PET	Positron emission tomography
PFS	Progression-free survival
PSA	Prostate-specific antigen
PSMA	Prostate-specific membrane antigen
SUVmax	Maximum standardised uptake value
SUVmean	Mean standardised uptake value
