# Supplementary material for: The Role of Dominant Lesion Site and SUVmax in the Depth of PSA Response in Metastatic Hormone-Sensitive Prostate Cancer
Source: Biomedicines. 2026 Sep 24;14(10):2160. doi: 10.3390/biomedicines14102160 (PMC13701217; doi:10.3390/biomedicines14102160)
Supplement: Supplementary file 1 [file biomedicines-14-02160-s001.zip › biomedicines-4542766-supplementary.pdf]

Supplementary Materials

The Role of Dominant Lesion Site and SUVmax in the Depth of PSA Response in Metastatic Hormone-Sensitive Prostate Cancer

Güner Akgüner, Ayşegül İlhan Güleşen and Özgen Ahmet Yıldırım

Table S1. Baseline characteristics of patients excluded at the six-month landmark compared with those included in the landmark analysis.

Variable	Excluded at landmark (n = 13)	Included (n = 92)	p
Age, median (IQR), years	71.0 (66.0–77.0)	72.0 (67.8–77.2)	0.486
Baseline PSA, median (IQR), ng/mL	99.5 (36.3–339.0)	128.0 (53.2–400.0)	0.466
De novo metastatic presentation, n (%)	11/13 (85%)	79/92 (86%)	1.0
Bone metastases, n (%)	11/13 (85%)	79/92 (86%)	1.0
Nodal metastases, n (%)	11/13 (85%)	70/92 (76%)	0.728
Visceral metastases, n (%)	3/13 (23%)	19/92 (21%)	1.0
Involved sites, median (IQR)	2.0 (2.0–2.0)	2.0 (1.0–2.0)	0.602
CHAARTED high volume, n (%)	8/13 (62%)	67/92 (73%)	0.512
LATITUDE high risk, n (%)	8/13 (62%)	56/92 (61%)	1.0
LDH, median (IQR), U/L	186.0 (160.0–260.0)	191.5 (168.2–239.2)	0.911
Haemoglobin, median (IQR), g/dL	13.6 (11.9–14.7)	13.1 (10.9–14.5)	0.647
Albumin, median (IQR), g/L	41.0 (40.0–44.9)	42.0 (38.5–44.5)	0.802
SUVmax, median (IQR)	15.8 (13.2–20.9)	17.3 (12.0–26.9)	0.931
Bone-dominant disease, n (%)	3/13 (23%)	31/87 (36%)	0.534
Deep PSA response, n (%)	5/13 (38%)	49/92 (53%)	0.382

IQR, interquartile range; LDH, lactate dehydrogenase. Thirteen patients were excluded from the six-month landmark analysis: four because a progression-free survival event occurred before the landmark and nine because follow-up ended before it. Seven of the nine censored patients had between five and six months of follow-up. Continuous variables were compared with the Mann–Whitney U test and proportions with Fisher's exact test. No difference reached significance, with the smallest p value being 0.382; this comparison has limited power given the small number of excluded patients and should not be read as establishing comparability.

Table S2. Baseline laboratory markers and depth of PSA response.

Marker	Deep vs. No Deep Response (median)	p	PFS Events, High vs. Low	p
LDH, U/L	185.0 vs. 202.0	0.062	14/52 vs. 3/53	0.008
Albumin, g/L	43.0 vs. 40.0	0.002	3/49 vs. 14/56	0.016
Haemoglobin, g/dL	13.7 vs. 12.4	0.018	7/51 vs. 10/54	0.257
Neutrophil-to-lymphocyte ratio	2.6 vs. 2.6	0.417	10/52 vs. 7/53	0.316
Platelet-to-lymphocyte ratio	152.2 vs. 138.3	0.703	9/52 vs. 8/53	0.744

Markers were compared between patients with and without a deep PSA response using the Mann–Whitney U test, and dichotomised at the cohort median for progression-free survival using the log-rank test. Only LDH retained an association with progression-free survival alongside depth of response in multivariable models; albumin and haemoglobin lost their associations once response depth was accounted for.

Table S3. Exploratory decomposition of the associations between bone dominance, depth of PSA response and progression-free survival.

Analysis	Estimate	95% CI	p
Step 1: total effect of bone dominance on PFS	HR 1.48	0.51–4.26	0.471
Step 2: bone dominance and deep PSA response	OR 0.25	0.10–0.62	0.003
Step 3: bone dominance, adjusted for response depth	HR 0.77	0.26–2.25	0.632
Step 3: response depth, adjusted for bone dominance	HR 0.057	0.007–0.45	0.0065
Exposure–mediator interaction	HR 0.59	–	0.54
Natural indirect effect (through response depth)	HR 1.62	1.22–2.22	< 0.05
Natural direct effect	HR 0.86	0.31–2.32	ns
Total effect (NDE × NIE)	HR 1.40	0.48–3.85	ns
E-value, response depth and PFS	19.5	4.2 at CI limit	–
E-value, bone dominance and response depth	7.3	–	–

HR, hazard ratio; OR, odds ratio; NDE, natural direct effect; NIE, natural indirect effect. Natural effects were estimated on the hazard-ratio scale within a counterfactual framework under the rare-outcome approximation, using a logistic model for the binary mediator and a Cox model for the outcome; confidence intervals derive from 4000 bootstrap resamples. The exposure-mediator interaction was not significant and the interaction-free model was therefore used. Because the total effect is null, the proportion mediated is undefined and is not reported. E-values quantify the strength of association an unmeasured confounder would need with both exposure and outcome to explain away the observed estimate. This decomposition rests on 15 events and on the assumption of no unmeasured confounding of the mediator-outcome relationship, which cannot be verified in these data; it is exploratory rather than confirmatory.

A simulation of collider stratification bias was additionally performed. An unmeasured common cause of mediator and outcome was introduced at increasing strengths (odds and hazard ratios of 2, 3, 4 and 5) under a true null direct effect. Conditioning on the mediator displaced the direct-effect estimate no further than a hazard ratio of 1.15, and in the opposite direction to that observed, indicating that collider bias cannot account for the reported pattern.

Table S4. Complete register of all 38 hypothesis tests performed, with unadjusted p values and Benjamini–Hochberg adjusted q values.

No.	Hypothesis	Domain	Test	p	q (BH)	Significant after FDR
1	–	Site	Ordered trend across sites (Spearman)	< 0.001	0.008	Yes
2	H1	PFS	Deep PSA response and PFS (six-month landmark)	< 0.001	0.010	Yes
3	–	Clinical	LATITUDE high risk and deep PSA response	< 0.001	0.010	Yes
4	H10	Site	Site of dominant lesion and deep PSA response	0.001	0.014	Yes
5	H7	Laboratory	Albumin and deep PSA response	0.002	0.016	Yes
6	–	Mediation	Bone dominance and deep PSA response (logistic)	0.003	0.016	Yes
7	–	Site	Bone dominance and deep PSA response	0.004	0.021	Yes
8	H4	Laboratory	LDH (median split) and PFS	0.008	0.033	Yes
9	–	Site	PSA nadir across sites (Kruskal–Wallis)	0.008	0.033	Yes
10	–	Clinical	CHAARTED high volume and deep PSA response	0.009	0.033	Yes
11	–	Site	Bone dominance and deep response, bone-metastatic subgroup	0.015	0.049	Yes
12	–	Laboratory	Albumin (median split) and PFS	0.016	0.049	Yes
13	H7b	Laboratory	Haemoglobin and deep PSA response	0.018	0.053	No
14	–	Clinical	LATITUDE high risk and PFS	0.021	0.055	No
15	–	SUVmax	SUVmax and LATITUDE risk group	0.022	0.055	No
16	–	Clinical	Bone metastases and deep PSA response	0.024	0.057	No
17	H2	PFS	Early (three-month) deep response and PFS (three-month landmark)	0.030	0.068	No
18	–	Clinical	Nodal metastases and deep PSA response	0.053	0.112	No
19	–	Clinical	Visceral metastases and PFS	0.062	0.123	No
20	–	SUVmax	SUVmax vs LDH (Spearman)	0.101	0.192	No
21	–	Clinical	Visceral metastases and deep PSA response	0.177	0.315	No
22	–	Clinical	CHAARTED high volume and PFS	0.182	0.315	No
23	H3	Treatment	ARPI agent and deep PSA response	0.200	0.330	No
24	H9	SUVmax	SUVmax and deep PSA response	0.284	0.449	No
25	–	SUVmax	SUVmax across sites (Kruskal–Wallis)	0.308	0.449	No
26	H5	Laboratory	NLR (median split) and PFS	0.316	0.449	No
27	–	Clinical	De novo presentation and deep PSA response	0.319	0.449	No
28	H8	SUVmax	SUVmax (log2) and PFS	0.375	0.493	No
29	–	SUVmax	SUVmax (median split) and deep PSA response	0.387	0.493	No
30	–	Clinical	De novo presentation and PFS	0.389	0.493	No
31	–	Clinical	Nodal metastases and PFS	0.445	0.546	No
32	H11	Site	Bone dominance and PFS (total effect)	0.468	0.552	No
33	–	SUVmax	SUVmax vs baseline PSA (Spearman)	0.479	0.552	No
34	–	SUVmax	SUVmax and CHAARTED volume group	0.631	0.687	No
35	–	Mediation	Bone dominance and PFS, adjusted for response depth	0.632	0.687	No
36	–	Clinical	Bone metastases and PFS	0.738	0.761	No
37	H6	Laboratory	PLR (median split) and PFS	0.744	0.761	No
38	–	SUVmax	SUVmax (median split) and PFS	0.761	0.761	No

Tests are ordered by unadjusted p value. Hypotheses H1-H11 were specified before the imaging analyses; the remaining tests are secondary or exploratory. All 38 tests were entered into a single Benjamini–Hochberg procedure, after which 12 remained significant.

Table S5. Comparison of excluded and analysed patients across baseline variables available in both groups.

Variable	Analysed (n = 105)	Excluded (n = 53)	p
SUVmax, median (IQR)	16.9 (12.3–26.8)	19.1 (11.8–30.0)	0.613
Evaluable baseline PET/CT, n	100	53	
Prostate, n (%)	39 (39%)	27 (51%)	0.293
Lymph node, n (%)	25 (25%)	14 (26%)	
Bone, n (%)	34 (34%)	12 (23%)	
Visceral or other, n (%)	2 (2%)	0 (0%)	
Enzalutamide, n (%)	45 (43%)	24 (46%)	0.497
Apalutamide, n (%)	17 (16%)	8 (15%)	
Abiraterone, n (%)	37 (35%)	14 (27%)	
Triplet regimen, n (%)	6 (6%)	6 (12%)	
Follow-up PET/CT performed, n (%)	49 (48%)	15 (28%)	0.025

IQR, interquartile range; PET/CT, positron emission tomography/computed tomography. Excluded patients had no recorded baseline clinical or laboratory data, so comparison was possible only for imaging, pathology and treatment variables. Continuous variables were compared with the Mann–Whitney U test and categorical variables with the chi-square or Fisher exact test; a single p value is given for each multi-category variable. Percentages for the site of the dominant lesion are calculated among patients with evaluable baseline PET/CT.

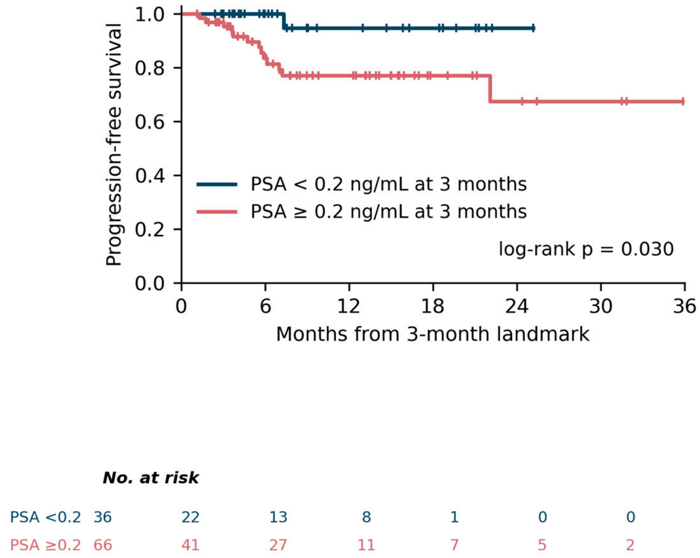

Figure S1. Progression-free survival by early (three-month) deep PSA response under a three-month landmark. Three patients with an event or censoring before the landmark were excluded (n = 102). Patients reaching a PSA below 0.2 ng/mL by three months progressed less often (1 of 36 versus 14 of 66), but the difference did not survive false-discovery-rate correction (q = 0.068) and this analysis is reported as hypothesis-generating. Censored observations are marked with a cross; numbers at risk are shown beneath the panel.

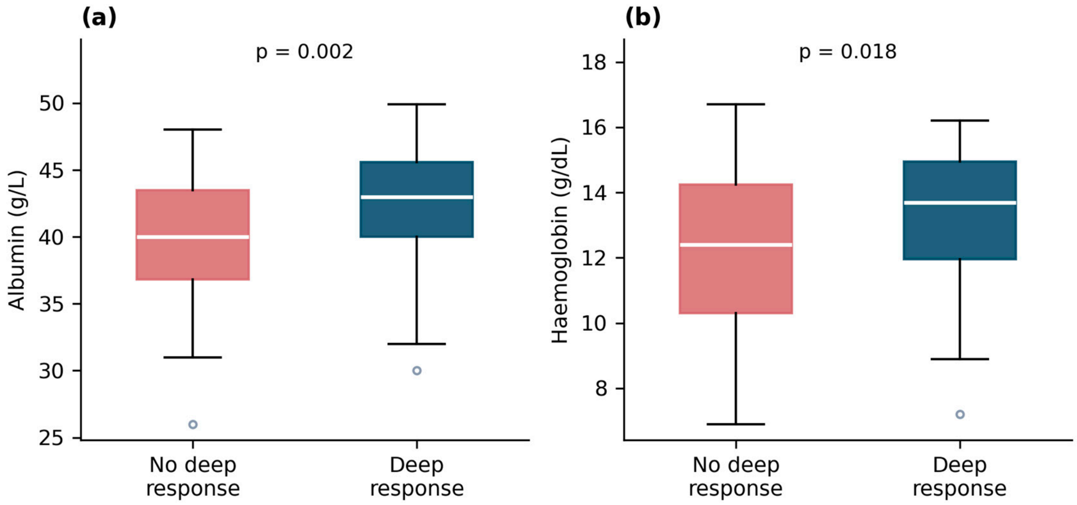

Figure S2. Baseline albumin and haemoglobin by depth of PSA response. Panels show albumin (a) and haemoglobin (b). Both were higher in patients achieving a PSA nadir below 0.2 ng/mL, although the haemoglobin difference did not survive false-discovery-rate correction. Circles denote outliers.
